# Supplementary material for: Content-rich biological network constructed by mining PubMed abstracts
Source: BMC Bioinformatics. 2004 Oct 8;5:147. doi: 10.1186/1471-2105-5-147 (PMC528731; doi:10.1186/1471-2105-5-147)
Supplement: Additional File 2 — The original results of the above study (non-essential files are deleted to keep the file size under the limit set by BMC bioinformatics). [file 1471-2105-5-147-S2.bz2 › chilibotAdditionalFile2/dip05/301ID7486683E1292/html/PLCG1_PDGFRB.html]

 


 **PLCG1** and **PDGFRB** 
  
Found 24 abstracts in PubMed, retrieved 05.  
 

 What does Google say? 
 PDF only 
| .edu only 

---

**Interactive relationship** (e.g. stimulation, inhibition, etc)

**Stimulatory relationship**- Autophosphorylation of beta PDGFR  [ **PDGFRB** ]  and tyrosine phosphorylation of PLC  [ **PLCG1** ]  gamma, PI3Kp85 and Shc were detected only in PDGF BB stimulated cells that express beta PDGFR  [ **PDGFRB** ] .  Ref: 12700668 Oncogene, 2003
**Neutral relationship**- Here we show that functional phospholipase C gamma1  [ **PLCG1** ]  PLC gamma1  [ **PLCG1** ]  is required for c Abl activation by PDGFR  [ **PDGFRB** ] .  Ref: 12652307 Nat Cell Biol, 2003

**Non-interactive relationship** (e.g. studied together, co-existance, homology, etc.)

- P< ; 0.0l, but the expression of PDGFR  [ **PDGFRB** ]  beta and PLC  [ **PLCG1** ]  activities did not show significant changes P>0.05.  Ref: 12589410 Sheng Li Xue Bao, 2001
- Activities of angiotensin converting enzyme ACE and aorta phospholipase C PLC  [ **PLCG1** ]  were tested, and levels of platelet derived growth factor receptor beta  [ **PDGFRB** ]  PDGFR  [ **PDGFRB** ]  beta and Galphaq 11 protein were measured by Western blot analysis.  Ref: 12589410 Sheng Li Xue Bao, 2001
- In comparison with the sham group, ACE activity, PLC  [ **PLCG1** ]  activity and the level of PDGFR  [ **PDGFRB** ]  beta were increased by 420.2 percent.  Ref: 12589410 Sheng Li Xue Bao, 2001
- PDGF receptor PDGFR  [ **PDGFRB** ] , protein kinase C alpha PKC alpha, phospholipase C gamma1  [ **PLCG1** ]  PLC gamma1  [ **PLCG1** ] , and PLD1 were examined by Western blot analysis.  Ref: 12086695 Exp Gerontol, 2002
- The protein levels of PKC alpha and PLC gamma1  [ **PLCG1** ]  were unchanged, but those of PLD1 and PDGFR  [ **PDGFRB** ]  were reduced with age.  Ref: 12086695 Exp Gerontol, 2002
